# Identification of transformation products from fluorinated lithium-ion battery additives TPFP**B** and TPFP**P**: forever chemicals of tomorrow?

**DOI:** 10.1007/s00216-024-05526-z

**Published:** 2024-09-13

**Authors:** Juliane Scholl, Jan Lisec, Hajo Haase, Matthias Koch

**Affiliations:** 1 Bundesanstalt für Materialforschung und -prüfung (BAM), Department of Analytical Chemistry and Reference Materials, Berlin, Germany; 2 Technische Universität Berlin, Department of Food Chemistry and Toxicology, Berlin, Germany

**Keywords:** HRMS, Simulation methods, Electrochemistry, Photochemistry, TOP assay, PFAS

## Abstract

Fluorinated organic compounds (FOCs) represent a class of synthetic chemicals distinguished by their resilient carbon–fluorine bonds, which demonstrate an ability to withstand environmental degradation over an extended period. The integration of FOCs into cutting-edge applications, including lithium-ion batteries (LiBs), presents considerable potential for environmental harm that has not yet been sufficiently addressed. This study focuses on the environmental fate of two fluorinated aromatics, tris(pentafluorophenyl)borane (TPFP**B**) and tris(pentafluorophenyl)phosphine (TPFP**P**), given their important role in improving the performance of LiBs. To achieve this, laboratory simulation methods including total oxidizable precursor assay, electrochemistry (EC), Fenton reaction, UV-C irradiation, and hydrolysis were employed. Liquid chromatography and gas chromatography coupled with high-resolution mass spectrometry were used for identification of transformation products (TPs) and prediction of their molecular formulae. Despite the structural similarity between TPFP**B** and TPFP**P**, distinct differences in electrochemical behavior and degradation pathways were observed. TPFP**B** readily underwent hydroxylation and hydrolysis, resulting in a wide range of 49 TPs. A total of 28 TPs were newly identified, including oligomers and highly toxic dioxins. In contrast, TPFP**P** degraded exclusively under harsh conditions, requiring the development of innovative conditioning protocols for EC. In total, the simulation experiments yielded nine structurally different compounds, including seven previously undescribed, partially defluorinated TPs. This study highlights the potential risks associated with the use of FOCs in LiBs and provides insight into the complex environmental behavior of FOCs.

**Graphical Abstract:**

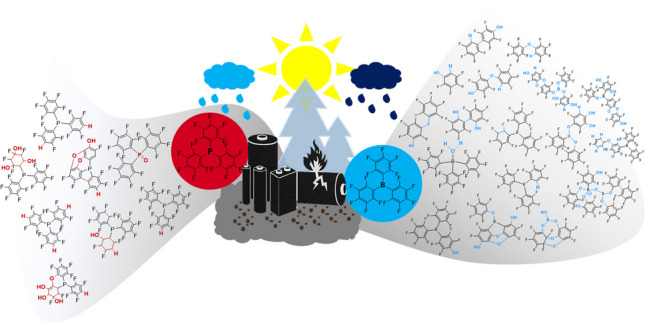

**Supplementary Information:**

The online version contains supplementary material available at 10.1007/s00216-024-05526-z.

## Introduction

Lithium-ion batteries (LiBs) have emerged as key components in modern society for portable energy storage and the ongoing energy revolution. Despite their extensive utilization, LiBs face serious challenges such as safety risks, capacity degradation, and the constant need for higher energy density. To address these issues, there is a growing focus on the use of fluorinated organic compounds (FOCs) as electrolyte components to improve the performance of LiBs [1–4]. Within LiBs, FOCs afford several advantages such as the design of high-polarity species with increased electronegativity and diminished viscosity. This results in enhanced solubility of lithium salts, improved electronic insulation, and increased chemical and oxidative resistance [4–8]. The first FOC integrated into LiBs was fluoroethylene carbonate (FEC), a fluorinated derivative of the base solvent ethylene carbonate. FEC is now a standard component of LiB electrolytes. Consequently, within LiBs, FOCs can be regarded as fluorinated analogues of base electrolytes such as ethylene carbonate. However, they also represent a class of entirely novel organic compounds with structural diversity, which typically incorporate molecular regions with increased electronegativity, such as fluorine, phosphate, and nitrile groups. F-mediated electron withdrawing properties also play a critical role in preventing free radical chain reactions by scavenging hydrogen radicals in fire and thus providing flame retardancy. Further, the C–F bond facilitates anode-mediated degradation, leading to effective passivation of anodes and cathodes and preventing further degradation of electrolyte components. This ultimately leads to improved cycle rates and stability, allowing for higher voltages and operating temperatures [2, 3, 9–11].

The introduction of novel industrial chemicals into a rapidly expanding field of applications always bears the potential that these chemicals enrich in wastewater or in terrestrial environments [12–15]. It has already been demonstrated that environmental processes have the potential to transform parent chemicals into more toxic and persistent transformation products (TPs), which often exhibit increased mobility [16, 17]. Increased mobility potentially facilitates the introduction and spread of TPs into previously unexposed ecosystems, thereby endangering a wider range of living organisms. Therefore, the transformation of chemicals often leads to unknown TPs with unknown properties. These considerations are also taken into account by the European Regulation on the registration, evaluation, authorisation and restriction of chemicals (REACH) [18], within exposure estimations including studies on possible transformations and environmental fates, despite the lack of clear guidance in this domain.

A prominent class of industrial chemicals is per- and polyfluoroalkyl substances (PFAS), which are closely related to FOCs due to their fluorine content. PFAS are distinguished by their exceptional persistence and are often referred to as “forever chemicals.” Some FOCs are actually PFAS according to the Organisation for Economic Co-operation and Development (OECD), due to a fully fluorinated methyl or methylene group [19]. Given their adverse impact on the environment and public health [20–22], PFAS should serve as a case study cautioning against the implementation of strategies for addressing FOCs that lack robust evaluation and monitoring. However, there is currently no established standard for investigating the environmental fate of industrial chemicals. Therefore, the scientific community has developed a number of reliable and rapid approaches to account for TP evaluation in risk assessment [16, 23–27]. One area of interest is the simulation of environmental transformation processes under laboratory conditions. These include, for example, electrochemistry (EC) and can be categorized as biotic or abiotic. Biotic processes are microbial degradation and metabolism, whereby only microbial degradation can be seen as a relevant environmental process for FOCs. Abiotic processes comprise chemical reactions, light-induced transformations (UV-A, UV-B, UV-C), and hydrolysis [16, 23, 28].

The simulation of chemical reactions often relies on hydroxyl radicals, which are commonly found in the environment and are known to react rapidly with industrial chemicals [29, 30]. One common method for simulating this process is through the Fenton reaction, where iron(II) ions catalyze the decomposition of hydrogen peroxide to form hydroxyl radicals [31]. Light-induced transformations involve exposing compounds to light of certain wavelengths, typically in the UV range between 100 and 400 nm. This can result in photolysis, which is divided into direct and indirect photolysis. Direct photolysis involves light energy absorbed by the parent compound to cause bond cleavage, whereas indirect photolysis produces reactive oxygen species that interact with the parent compound and cause its transformation [32, 33]. Hydrolysis is a significant environmental process, as it produces increased water-soluble TPs. However, the investigation of TPs generated by hydrolysis has been relatively underrepresented in previous research [34–38]. Electrochemical approaches even include the ability to mimic various biotic and abiotic processes, which were successfully shown [39–43]. In EC-based TP generation, parameters such as working electrode material, pH value, solvent, and electrolyte can be easily modified, rendering it a valuable tool in research on environmental transformation behavior and persistence [28, 39, 40, 44]. At present, no studies have been conducted on TP simulation of relevant FOCs from LiBs, so that their fate is difficult to predict, which in turn poses a potential environmental risk.

FOCs proved to be very stable compared to conventional TP-generating methods. To challenge this stability and investigate whether it is comparable to highly persistent substances such as PFAS, we borrowed a method used in PFAS analysis, the total oxidizable precursor (TOP) assay. During thermolysis of persulfate, hydroxyl radicals are formed under conditions of high pH [45]. This harsh environment results in the conversion of the initial compounds into what are referred to as “end products” or, alternatively, to their complete degradation [46]. The method makes use of the distinctive stability of PFAS, either for the conversion of PFAS into carboxylic acids, which represent the final products of the process, or for the retention of the original carboxylic acids. Nevertheless, the result of this process does not mimic environmental conditions, but this method could be used to determine the environmental persistence of the investigated substances in comparison to PFAS.

**Fig. 1:**
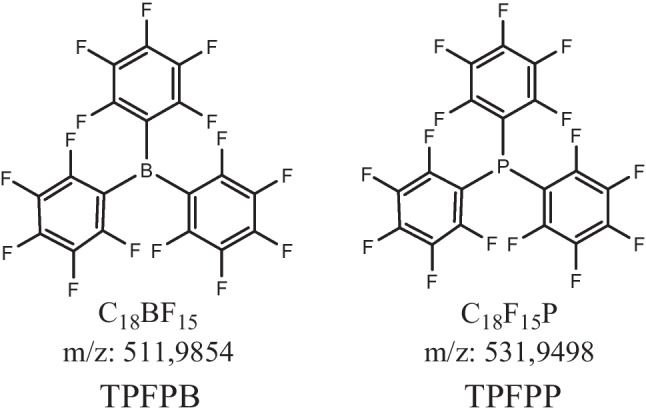
Chemical structures, exact molecular mass, and chemical sum formula of TPFP**B** and TPFP**P**

To study the environmental fate of FOCs, two relevant model compounds, tris(pentafluorophenyl)borane (TPFP**B**) and tris(pentafluorophenyl)phosphine (TPFP**P**), have been selected (Fig. 1). Although they are commonly used in LiBs, no TP data exist to date.

The incorporation of TPFP**B** and TPFP**P** into LiBs has been demonstrated to enhance performance in a number of ways. TPFP**B** has been reported to improve thermal stability and cycling performance, and to enable high-voltage applications due to significant solid electrolyte interphase (SEI) improvement [47–54]. TPFP**P** has also been shown to contribute to SEI formation, enabling high-voltage applications [55, 56], and additionally to possess flame-retardant properties [57]. Li et al. in 2020 observed similar properties for both compounds when added alternately in the same experimental setup [53]. In the environmental degradation process, the fluorinated aryl structures of TPFP**B** and TPFP**P** may play a key role, as their high electron density in the aromatic system, which makes them stable, is further enhanced by the presence of the fluorine atoms. The strong positive mesomeric effect of fluorine on the ring carbon hinders nucleophilic attack, emphasizing the importance of fluorine elimination in degradation processes to avoid the formation of toxic “dead-end” TPs that are not further degraded [58, 59].

Although the two compounds share these similar structural features, they exhibit different chemical behaviors due to their different central atoms, which make TPFP**B** a strong Lewis acid and TPFP**P** a Lewis base. The different electron density effects of Lewis bases and acids in these compounds may have an impact on their susceptibility to degradation. Lewis bases can potentially increase electron density while Lewis acids can decrease it. This may make TPFP**B** more susceptible to degradation of fluorinated aromatic ring structures. The processes and risks involved in the transformation process remain unknown.

To close the knowledge gap, environmental simulation methods using Fenton reaction, UV-C irradiation, hydrolysis, and EC-based approaches were performed to study the degradation behavior of TPFP**B** and TPFP**P**. The TOP assay was selected to evaluate reactivity and persistence in the environment compared to PFAS, and for simulation of light-induced transformations, ionizing UV-C irradiation (200–280 nm) was utilized to evaluate transformation because of the proposed high stability of the compounds. Liquid chromatography (LC) and gas chromatography (GC) coupled with high-resolution mass spectrometry (HRMS) were used to identify the resulting TPs. Our results provide valuable first insights into the environmental fate and potential impact of FOCs, helping to develop appropriate risk assessment and management strategies.

## Materials and methods

### Chemicals

TPFP**B** (97%) and TPFP**P** (95%) were purchased from Fisher Scientific GmbH (Schwerte, Germany). All solvents used for LC were MS grade and those used for GC were p.a. grade. The LC–MS solvents including acetonitrile (ACN) and methanol (MeOH) and the modifier ammonium acetate (NH_4_Ac) were obtained from TH. Geyer GmbH & Co. KG (Renningen, Germany). Formic acid (FA) was obtained from Carlo Erba Reagents GmbH (Emmendingen, Germany), while ultrapure water (0.055 µS at 25 °C) was produced with a Purelab Flex 2 from Elga Veolia Water Technologies (Celle, Germany). Sodium chloride (NaCl) for extraction procedures and GC solvents acetone (Ac), ethyl acetate (EtOAc), and tert-butyl methyl ether (MtBE) were also obtained from TH. Geyer GmbH & Co. KG, together with TOP assay reagent potassium peroxydisulfate (99.0%), sodium hydroxide (NaOH, 30%), and hydrochloric acid (HCl, 35–38%). Fenton reagent hydrogen peroxide 30% w/w in H_2_O puriss., stabilized, was provided by Sigma-Aldrich (Taufkirchen, Germany), iron(II) sulfate heptahydrate by Sigma-Aldrich (Seelze, Germany), and manganese(IV) oxide (98%) by Carl Roth GmbH + Co. KG (Karlsruhe, Germany).

### Simulation methods

#### TOP assay

For the TOP assay, 0.5 mL of 10 mM FOC dissolved in EtOAc (TPFP**B**) or Ac (TPFP**P**) was added to a 20 mL headspace vial, the solvent was evaporated, 12 mL water was added, 1.2 g of persulfate was weighed in, and 1.2 mL of NaOH 30% was added. The vials were sealed, heated to 85 °C for 7 h using a drying cabinet (Memmert GmbH + Co. KG, Schwabach, Germany), and agitated manually every hour. To stop the reaction, the vials were cooled to 4 °C in a refrigerator and the pH was checked using pH test paper. For LC–MS/MS measurement, the pH was adjusted to pH < 5 using concentrated HCl (35–38%), an aliquot was taken and extracted with 4 mL Ac/ACN (50/50, v/v), saturated with NaCl, shaken for 10 min, and the aqueous phase discarded.

#### Fenton reaction

Fenton reaction was carried out in water containing 100 μM TPFP**B** or 2 mM TPFP**P**, 1 mM iron(II) sulfate heptahydrate and 12 mM H_2_O_2_, resulting in a total volume of 1 mL. The pH was adjusted to 3 by adding 1 µL concentrated HCl. The mixture was shaken at 900 rpm at 25 °C using a thermo shaker (HTA-BioTec, Bovenden, Germany). The reaction was stopped by adding 10 μL of 300 mM manganese dioxide after different time intervals (60, 120 min). For analysis, a 0.3 mL aliquot of the reaction solution was extracted with 2 g NaCl, 0.4 mL MtBE, shaken for 10 min, and the aqueous phase discarded.

#### UV-C irradiation

Transformation was performed in a quartz UV reactor equipped with a TQ 150 W medium-pressure mercury lamp (Heraeus Noblelight GmbH, Hanau, Germany) emitting light in the wavelength range λ 280 to 200 nm and cooled to a constant temperature of 15 °C by an internal cooling system. UV irradiation was performed after filling the UV reactor with 200 mL solution of 50 µM TPFP**B** in MeOH/H_2_O (4:1, v/v) or 0.2 mM TPFP**P** in Ac/MeOH/H2O (31:56:14, v/v/v). The samples were stirred constantly at 800 rpm with a magnetic stirrer, and 2 mL aliquots were collected at different time points (0, 30, 60 min). For analysis, the samples were measured either directly or after extraction (see Fenton reaction).

#### Hydrolysis

For hydrolysis, 0.1 mL of 10 mM FOC stock solution in ACN (TPFP**B**) or Ac (TPFP**P**) was added to headspace vials, and the solvent was completely desiccated. Afterwards, 11 mL H_2_O and 1.3 mL NaOH (30% w/w) for basic conditions, or 1.3 mL concentrated HCl for acidic conditions, were added. Vials were capped, heated to 85 °C for 7 h using a drying cabinet (Memmert GmbH + Co. KG, Schwabach, Germany), and manually shaken every hour. To stop the reaction, the vials were cooled to a temperature of 4 °C in a refrigerator and the pH was adjusted to approximately 2–3 using pH testing paper. An aliquot was taken and extracted (see Fenton reaction).

##### EC

Dilutions of TPFP**B** and TPFP**P** were injected into the μ-PrepCell™2.0 (Antec Leyden) electrochemical flow-through cell, equipped with a boron-doped diamond electrode (BDD) as working electrode, a HyREF™ reference electrode (Pd/H2), and a black polymeric auxiliary electrode (Conductive PEEK (PEEK, 30% carbon fiber-reinforced). Electrochemical transformation was performed with the Roxy™ potentiostat (Antec Leyden, Zoeterwoude, Netherlands) at a flow rate of 50 µL/min using a syringe pump (11 Plus dual, Harvard Apparatus, USA). Samples were collected for offline analysis. Prior to each experiment, the electrodes were cleaned and activated using pulse programs supplied by Antec.

All simulations were conducted independently in triplicate (EC, hydrolysis, Fenton, TOP assay) or in duplicate (UV-C). These are referred to as simulation sets.

### Mass spectrometry measurements

#### TPFP**B**

TPFP**B** does not vaporize undecomposed and is soluble in polar solvents, rendering it suitable for LC–MS based measurements. TPFP**B** and corresponding TPs were separated and detected with an Agilent 1290 Infinity II (Agilent Technologies, Waldbronn, Germany) connected to an electrospray ionization (ESI) source with a TripleTOF^®^ 6600 Quadrupole (Sciex, Darmstadt, Germany). The system was controlled by Analyst^®^ TF1.8.0 (AB Sciex). Tuning was performed prior to measurement to gain mass accuracy < 2 ppm. The recorded mass range was 100–3000 Da in negative ion mode, using a source temperature of 300 °C and recording MS/MS scans in information-dependent acquisition (IDA) mode. For chromatographic separation, 5 μL was injected into an Agilent Zorbax Eclipse XDB C18 column (2.1 mm × 100 mm, particle size 1.8 μm) heated to 40 °C. The mobile phase consisted of 0.1% FA aqueous solution and ACN as mobile phases, starting with 80% water held for 1 min, increased to 99% ACN within 8 min and held for 3.9 min. This was followed by a decrease back to 80% water within 0.1 min and held for 3 min. The flow rate was set at 0.5 mL/min.

#### TPFP**P**

TPFP**P** is very weakly ionized by ESI. Its TPs are not ionized at all and are only soluble in nonpolar solvents, which required us to conduct GC measurements and independent analysis of the two compounds. To this end, TP identification and relative abundance determination were performed on a gas chromatograph coupled to low-resolution mass spectrometry (GC–LRMS) with nominal mass resolution. The Agilent 7890A gas chromatograph was coupled to an Agilent MSD 5975C electron impact ionization mass spectrometer, equipped with an Agilent J&W DB-5ms UI thin film column (15 m × 180 μm × 0.18 μm). Helium was used as the carrier gas at a flow rate of 1.95 mL/min, and the oven was programmed at 45 °C for 3 min, heated with 13 °C/min to 130 °C and held for 0 min, then 19 °C/min to 325 °C and held for 4 min, while the transfer line was set at 325 °C. The measurement was performed with an ion source temperature of 230 °C and a mass range of 35–1050 m/z in positive mode. A Gerstel cooled injection system was programmed to 35 °C with a hold time of 0.1 min, and then to 320 °C and set to hold for 6 min at 9 °C/s.

Determination of the exact masses of the TPs identified by GC–LRMS was performed using the Agilent 7200 gas chromatography–quadrupole time-of-flight mass spectrometry (GC-QTOF-MS) system, equipped with an ultra-inert GC column, J&W HP-5ms (30 m × 250 μm × 0.25 μm). The oven was programmed to 45 °C and held for 2 min, then heated at 19 °C/min to 325 °C and held for 4 min, while the transfer line was set to 325 °C. The measurement was conducted at an ion source temperature of 230 °C. A 1 μL volume of liquid sample was injected splitless at 300 °C using an Agilent single taper, Ultra Inert. The system was controlled by MassHunter Workstation version 10.0 (Agilent Technologies, Inc.). A spectral library search using the Mass Spectral Library (NIST 17) was performed to identify the TPs.

The GC-QTOF-MS procedure involves electron ionization (EI). Given that the standard ionization energy of 70 eV is a hard ionization technique with extensive fragmentation to achieve robustness and sensitivity, this often results in a lack of molecular masses within spectra necessary for identification of unknowns. To address this issue, low-energy electron ionization (LE-EI) has been demonstrated to result in mass spectra with less fragmentation, which can be used in the identification of unknowns [60–62]. To perform an unknown analysis by GC-QTOF-MS, EI spectra were obtained using an electron energy of 70 eV, as well as with LE of 18 eV. Prior to the measurement, QTOF was calibrated to a mass accuracy of less than 1 mDa.

### Data processing

#### TPFP**B**

The LC-QTOF data were processed using MSDIAL version 4.9.221218, and the resulting peak list was exported. For statistical analysis, an in-house script was developed using R 4.3.0 and RStudio 2023.06.0–421 (SI-1.2). The script filters components based on minimum peak intensities and intensities relative to blanks and controls, as well as their presence in two replicates of a simulation set. Components were checked for specific boron isotope patterns in raw data. A merge step was implemented to resolve repeated annotations (Figure SI-1.1) and to resolve annotations of masses related to the same compound generated in the ion source. Peak grouping according to retention time was implemented (Figure SI-1.2 to 4) to resolve the issue of multiple annotations for a single compound. To define the molecular mass and calculate the chemical sum formula suggestions, the mass with the highest intensity in the isotopic spectrum was selected. Chemical sum formula suggestions were calculated using restricted elements (C, F, N, H, O), and five additional rules were implemented to minimize the number of inconclusive chemical sum formula suggestions. This was achieved by setting a threshold of the smaller of two values, 3.5 mDa or 3.5 ppm, respectively. Conclusive structural formulas were then manually analyzed and assigned a confirmation level (CL). A detailed description of the script development is provided in the Supporting Information (SI-1).

#### TPFP**P**

GC-LRMS data were processed using Agilent MassHunter Qualitative Analysis software. Peak picking and subtraction of false-positive peaks were performed manually in overlay mode. The exact masses and EI spectra of the identified TPs were obtained in a GC-QTOF measurement. The QTOF data were observed for molecular masses. The molecular masses were used to calculate possible sum formulas within a threshold of ±5 ppm and using restricted chemical elements (C0-30; H0-10; P0-20; F0-20; O0-20). A manual review of the calculated molecular formula proposals was conducted to determine conclusive structural formulas. Isotope abundance and MS/MS data underwent manual analysis to confirm the conclusive structural formulas and set the CL.

## Results and discussion

### Optimizing electrochemical conditions to allow high voltage

To conduct electrochemical investigations on the two LiB additives, it was first necessary to optimize the experimental conditions. The optimization parameters included pH, solvents, electrolytes, electrodes, and modifiers. The ideal parameter values are shown in Table 1. A complete enumeration of all EC parameters utilized for the transformation of TPFP**B** and TPFP**P** is provided in the Supporting Information (SI-2). BDD was selected for its extensive potential range and minimal background interference [63].

**Table 1:** EC parameters developed for transforming TPFP**B** and TPFP**P**, using the μ-PrepCell equipped with BDD electrode

Substance (conc.)	Solvent	Modifier/electrolyte	Method ID
TPFP**B** (10 µM)	ACN/H_2_O (99.7:0.3 v/v)	1% FA	EC_ox_, EC_red_
TPFP**P** (20 µM)	ACN/EtOAc/H_2_O (65:35:0.3 v/v)	1% FA	EC_ox_
TPFP**P** (20 µM)	MeOH/Ac (65:35 v/v)	5 mM NH_4_Ac	EC_ox2_

One of the primary challenges encountered was the limited solubility of TPFP**P** in conventional EC solvents, including ACN, MeOH, and H_2_O, requiring the use of non-polar solvents and solubilizing solvents to prevent precipitation. Another considerable obstacle was the low and absent transformation (TPFP**B** and TPFP**P**, respectively) within the BDD potential range (–2.5 V to +3.5 V). This range is based on water electrolysis. Consequently, the level of water was constrained to mitigate the risk of electrolysis and to expand the potential window while maintaining environmental conditions. The resulting potential ranges for TPFP**B** were 0.0 to 4.1 V and −4.0 to 0.0 V, while for TPFP**P** the ranges were 3.5 to 4.1 V and −4.1 to −2.5 V. The potentials were ramped at a velocity of 20 mV/s and the experiments were conducted offline to account for the different detection techniques required for each substance.

In conclusion, these previously unreported conditions allow for extended potentials while maintaining environmental conditions. The need for high potentials was expected, given the known voltages (up to 4.2 V) that LiB electrolytes are subjected to in practice.

### Identification and annotation of 49 TPFP**B**-derived TPs from several degradation pathways

Various laboratory simulation methods were utilized to mimic the environmental degradation of the LiB additive TPFP**B**. As an outcome of the investigation, a total of 49 different TPs were identified, of which 29 were conclusively identified by their sum formulae (Table 2, SI-3) and 28 were identified for the first time.

**Table 2:** Compilation of the results of the different simulation methods employed on TPFP**B**. The analysis was performed using LC-QTOF (negative mode). Compounds are considered confirmed if they were detected in at least two replicates of a simulation set. The proposed chemical formulae, intensities, and confirmation level (CL) are displayed for each compound. Adducts are often formed including formic acid (FA, HCOOH) or acetonitrile (ACN, CH_3_CN). The intensities are indicated relative to the strongest signal (vs: very strong) > 60%, (s: strong): 40–60%, (m: moderate): 20–40%, (w: weak) 10–20%, (vw: very weak) < 10%). Abbreviations of the simulation methods: EC (oxidative and reductive conditions); EC_ox_ (EC oxidative conditions); EC_red_ (EC reductive conditions); UV (UV-C irradiation); Fe (Fenton reaction); H (hydrolysis with basic and acidic conditions); H_b_ (hydrolysis alkaline conditions); H_a_ (hydrolysis acidic conditions)

TP-ID	MASS MEAS	MASS CALC	FORMULA	ADDUCT	INTENSITY	CL	SIM. METHOD
1	164.9970	164.9969	C_6_HF_4_O	[M-H]^−^	vw	5	H_b_
2	209.9800				vw		H_b_
3	244.9040				vw		H_b_
4	312.9870	312.9905	C_12_H_2_F_8_O	[M-H]^−^	vw	3	H_b/_EC_red_
5	328.9820	328.9854	C_12_H_2_F8O_2_	[M-H]^−^	vw	5	H_b_
6	330.9830	330.9811	C_12_HF_9_O	[M-H]^−^	vw	5	H_b_/EC_red_
7	366.0010	366.0007	C_12_HF_7_O_3_	[M + ACN-H]^−^	vw	5	EC_red_
8	370.0050	370.0053	C_12_H_2_F_8_O_2_	[M + ACN-H]^−^	vw	5	EC_red_
9	382.9910	382.9906	C_12_BF_12_	[M]^−^	vw	5	EC_red_
10	432.9760	432.9771	C_12_HBF_8_O_5_	[M + FA-H]^−^	vw	3	EC
11	493.0080	493.0075	C_18_H_4_BF_13_O	[M-H]^−^	vw	4	UV/H/Fe
12	502.9790				vw		EC_red_/EC_ox_
13	510.9970	510.9981	C_18_H_3_BF_14_O	[M-H]^−^	vw	4	UV/Fe/H
14	520.9860	520.9860	C_18_H_3_BF_12_O_4_	[M-H]^−^	vw	4	EC_ox_
15	522.9800	522.9817	C_18_H_2_BF_13_O_3_	[M-H]^−^	vw	4	Fe/EC_red_/EC_ox_
16	524.9870	524.9834	C_18_H_2_BF_9_O_5_	[M + FA-H]^−^	vw	3	Fe/EC_red_/EC_ox_
17	525.0030				vw		EC
18	526.9920	526.9930	C_18_H_3_BF_14_O_2_	[M-H]^−^	vw	4	Fe/H
19	528.9870	528.9886	C_18_H_2_BF_15_O	[M-H]^−^	vs(EC_ox_)/ s(EC_red_)	3	Fe/H/UV/EC
20	538.9940	538.9930	C_18_HBF_14_	[M + FA-H]^−^	vw	3	EC/UV
21	541.0020				vw		UV
TPFP**B**	543.0040	543.0037	C_18_BF_15_	[M + H_3_COH-H]-	w	1	UV
22	547.0190	547.0192	C_18_H_7_BF_14_O_3_	[M-H]^−^	vw	4	EC_red_
23	548.0030				vw		EC_red_
24	548.0160				vw		EC_ox_
25	548.9840				vw		EC
26	550.0090	550.0090	C_18_HBF_14_O	[M + ACN-H]^−^	vw	4	UV/EC_red_
27	550.9860				vw		EC
28	552.9940				vw		EC_ox_
TPFP**B**	556.9880	556.9836	C_18_BF_15_	[M + FA-H]^−^	vw	1	
29	563.9990				vw		EC/UV
30	564.0090	564.0082	C_18_H_2_BF_13_O_3_	[M-H]^−^	vw	3	EC
31	566.0220	566.0239	C_18_H_4_BF_13_O_3_	[M + ACN-H]^−^	vw	3	EC_ox_
32	569.0380	569.0388	C_24_H_8_BF_13_O	[M-H]^−^	vw	3/4	UV
33	570.9980				vw		Fe
34	584.9870				vw		EC
35	586.0000				vw		EC_ox_
36	586.9870				vw		EC_ox_
37	590.9490				vw		EC_ox_
38	592.0090	592.0067	C_18_H_4_BF_11_O_7_	[M + ACN-H]^−^	vw	3/4	EC_ox_
39	594.0230	594.0224	C_18_H_6_BF_11_O_7_	[M + ACN-H]^−^	vw	3/2b	EC_ox_
40	602.9760				vw		EC_ox_
41	612.0140	612.0129	C_18_H_5_BF_12_O_7_	[M + ACN-H]^−^	vw	3	EC_ox_/Fe
42	613.0220				vw		Fe
43	626.0100				vw		EC_ox_
44	634.9350				vw		H
45	658.9830	658.9813	C_24_H_5_BF_12_O_8_	[M-H]^−^	vw	3	Fe/UV/H
46	662.9740	662.9726	C_24_H_3_BF_14_O_6_	[M-H]^−^	vw		EC_red_
47	708.9640				vw		EC_red_/Fe
TPFP**B**	1058.9830	1058.9456	C_36_H_4_B_2_F_30_O_2_	[2 M + 2 H_2_O-H]^-^	vw		Fe/H/UV/EC
TPFP**B**	1068.9660	1068.9689	C_36_B_2_F_30_	[2 M + FA-H]^−^	vw	2b	Fe/H/UV/EC_ox_
48	1080.9560	1080.9550	C_36_H_2_B_2_F_27_O_7_	[M-H]^−^	vw	4	Fe/H/UV
49	1136.9640				vw		EC

The confidence in identifying unknown compounds through HRMS techniques was evaluated using the categories suggested by Schymanski et al. [61]. Here, annotation confidence is based on analytical information including exact mass, isotope pattern, availability and conclusiveness of MS/MS data, library matches, rt comparison, and reference standards. Level 1 is the most reliable, as it requires confirmation by an analytical standard [64]. However, as no analytical standards are available for novel compounds, level 2b was the highest possible assignment for the identified TPs in this study. At level 2b and above, no structural isomers are possible. The annotation levels were developed for the non-targeted analysis of complex mixtures. Hence, their requirements can be considered as too strict in this work, as the possible chemical space for de novo annotations is highly restricted by the chemical elements of the precursor molecules. Nevertheless, we apply these levels to allow for insights into the possibility of structural isomers and to assess the reliability of the MS and MS/MS data.

**Fig. 2:**
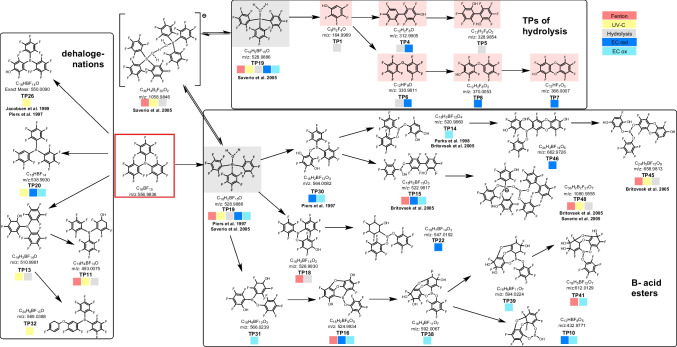
Proposed structures of the TPs and transformation pathways from the different simulations of TPFP**B**. Numbering is according to masses (see Table 2). Literature references are given to substances with structural similarity. Red background: indicates toxicological relevance; gray background: indicates high occurrence

Further validation could be obtained from the observation that numerous TPs were generated by multiple simulation methods. TP 19 emerged as the most prevalent product across all simulations, while certain methods showed near-complete conversion. The presence of other TPs was detected at low levels. The TOP assay simulation method yielded no results, as no TPs were detected following the simulation. This indicates that the TOP assay resulted in complete decomposition of the substance into metabolites not detectable by LC and GC–MS. Figure 2 depicts the proposed structures of the TPs and the transformation pathways. For illustration, one likely constitutional isomer was selected, as MS/MS spectra did not always provide distinctive formulas for derived TPs as implied by the CL (Table 2).

#### Modifications

Transformation of TPFP**B** resulted in the formation of a diverse array of products, including defluorination products, hydroxylation products, boronic acid esters, dioxin structures, diphenyls, diphenyl ethers, and oligomers. Many of these compounds were previously undocumented. To gain insight into the formation of some TPs, references to structurally similar compounds in Fig. 2 were employed. For example, Piers et al. and Jacobsen et al. reported adducts with nitriles [65, 66], while Di Saverio et al. described the coupling of B–O–B [67]. Furthermore, Britovsek et al. demonstrated the synthesis of borinic and boronic acid esters (B–O–Ar) [68].

#### Hydrolysis

Hydrolysis of TPFP**B** under alkaline conditions yielded a series of products lacking boron, as confirmed by EC under reductive conditions. The primary products are a fluorinated phenol, fluorinated diphenyl ethers (TP 6, TP 8), fluorinated diphenyls (TP 4, TP 5), and even a fluorinated dioxin formed with EC (TP 7). Chen et al. also observed the formation of C–C bonds with fluorinated phenols through electrophotocatalysis [69]. TP 19, the primary product of all simulations, is formed by hydrolysis. This process has been documented for TPFP**B** [65, 67]. The process commences with the reaction between boron and water, which ultimately results in the gradual release of C_6_F_5_H, in agreement with the findings of Piers et al. [65]. The released compound may subsequently react to TP 1 through hydroxylation and F release. This discrepancy could be attributed to either a rapid reaction or a limitation of the analytical detection method, potentially due to ionization or solubility constraints.

#### Defluorination

Dehalogenation reactions were observed in a number of TPs (e.g., TP 26, TP 20, TP 13, TP 11, TP 32). The formation of these TPs was predominantly achieved through UV-C irradiation. For instance, TP 20 was produced via the hydrodefluorination process, involving the photoheterolysis and hydrogen abstraction from a polar solvent [59, 70, 71], which is considered direct photolysis [72]. Indirect photolysis involves the generation of reactive oxygen species that react with the substrate, as observed with TP 26. This process was also documented by Arnold in 2020 [73]. Comparable products are generated through hydrolysis and the Fenton reaction (TP 45, TP 46, TP 48). Another potential reaction pathway which can be considered leads to the formation of compounds such as TP 11, TP 13, TP 32, and TP 18. This pathway involves the electrophilic addition of water to the double bond of the aryl group.

#### Boronic acid esters

A number of TPs indicate the formation of boronic acid esters. Similar compounds were reported by Britovsek in 2005 [68], but via a different synthesis from a different precursor. In this case, C–B bonds have to be hydrolyzed first, as observed for TP 10. As described by Brooks et al. [74, 75], the released aryl groups may form phenols, which subsequently react with boronic acids to form esters. Oligomerization may occur, as observed in previous studies, with the formation of [B–O–B] bonds within the oligomers, known as diboroxanes [67, 74, 76].

#### Oligomers

The formation of oligomers (TP 48, TP 45, TP 46), including structures analogous to those of dioxin, diphenylethers, and even diboroxanes (TP 48), has been observed in the course of a number of different simulations. In the environment, oligomers exhibit significantly altered chemical fate. They are reduced in mobility and water solubility, and potentially changed in leachability and biological activity. This immobilization prevents the substances from leaching into groundwater and instead incorporates them into soil organic matter. However, biotic alterations could alter the substances again and lead to modified substances that would have a delayed health risk, such as phytotoxic effects, impacts on soil organisms, or uptake by crops [77, 78].

#### Electrochemistry

The highest number of TPs was formed by EC, although confirmation of related structures was not consistently achieved by secondary simulation methods. Nevertheless, these findings make EC an interesting screening method for the simulation of several abiotic processes. Furthermore, the transformation of TPFP**B** produced TPs containing many of the previously described alterations (e.g., TP 41, TP 15, TP 48, TP 45). These TPs comprise dioxin structures, diphenyl structures, boronic acid esters, hydroxylation, and defluorination. However, due to the complex and novel nature of these substances, a toxicological or environmental assessment of these specific TPs is not feasible. Nevertheless, for certain structural equivalents, general extrapolations may apply.

#### Toxicologically relevant equivalents

The halogenated equivalents of the TPs formed by alkaline hydrolysis are highly toxic compounds such as polybrominated diphenyl ethers, polybrominated biphenyls, and dioxins (polychlorinated dibenzo-**p**-dioxins), which are regulated under Annexes A and C of the Stockholm Convention on Persistent Organic Pollutants [79]. The formation of dioxins or dioxin-like compounds is a major concern due to their high toxicity, persistence, environmental distribution, bioaccumulation in all living organisms, and carcinogenic potential. Compared to their brominated or chlorinated analogues, fluorinated dioxins show less toxicity but pose a greater risk to the immune system [80, 81]. Conversely, one study found fluorinated phenols to be more toxic than other halogenated analogs [82].

#### Electrochemistry for biotic processes

This study has focused on the abiotic formation of TPs. Another significant mechanism, namely, biotic transformation in the environment, was not considered, but EC has also been demonstrated to simulate biotic processes in several studies [42, 83–85]. Existing literature indicates the potential for biotic degradation of fluorinated aromatics through enzymatic removal of a fluorine by hydroxylation, resulting in the formation of phenols, catechols, hydroquinones, and trihydroxyfluorobenzenes [58, 86, 87]. The degradation of these compounds ultimately results in the formation of two end products: maleic acid and muconolactones. It is noteworthy that our study additionally identified hydroxylation and dihydroxylation products (TP 26, TP 31, TP 14) for TPFP**B**, which may reflect biotic processes.

### EC and UV-C treatment leads to several novel TPs of TPFP**P**

The environmental impact of the TPFP**P** additive utilized in LiBs was investigated through a series of laboratory simulations. The investigation identified a total of nine distinct TPs of TPFP**P**, eight of which were conclusively identified by their respective sum formulas (see Table 3 and SI-3). Of these, seven are reported here for the first time.

**Table 3:** List of TPs of TPFP**P** generated from simulation methods, including suggested chemical formulas, intensities, CL, and simulation technique; TP order is according to molecular masses. Analysis was performed by GC-LRMS and GC-QTOF measurement (70 eV and 18 eV). If detected in two replicates of a simulation set, a confirmation was assumed. Intensity values are derived from a ratio relative to the highest signal, which is typically the precursor (vs: very strong) > 60%, (s: strong): 40–60%, (m: moderate): 20–40%, (w: weak) 10–20%, (vw: very weak) < 10%). Simulation methods are indicated by abbreviations: EC: oxidative conditions EC_ox_: NH_4_Ac; EC_ox2_: FA; UV (UV-C irradiation); Fe (Fenton reaction); Ha (hydrolysis acidic conditions). * TP molecule ion: 567.9928 (at 18 eV) and 554.9994 (at 70 eV)

TP-ID	MASS MEAS	MASS CALC	FORMULA	INTENSITY	CL	SIM. METHOD
1	411.9731	411.9724	C_18_H_3_F_6_O_3_P^+^	w(EC_ox_)/ vw(UV)	3	EC_ox_/UV
2	477.9796	477.9775	C_18_H_3_F_12_P^+^	vw	3	EC_ox_/UV
3	495.9701	495.9681	C_18_H_2_F_13_P^+^	vw	3	EC_ox_/UV
4	507.9901	507.9922	C_18_H_7_F_10_O_4_P^+^	vw	3	EC_ox_
5	513.9606	513.9587	C_18_HF_14_P^+^	vw	3	EC_ox_/UV
6	520.0259	520.0256	C_18_H_10_F_13_OP^+^	vw	4	EC_ox_
TPFP**P**	531.9507	531.9493	C_18_F_15_P^+^	vs	1	
7	547.9453	547.9442	C_18_F_15_PO^+^	vs(EC_ox2_)/ vw(H_a_, Fe)	2a	Fe/EC_ox2_/H_a_
8	555.9941			vw		EC_ox_
9	567.9928*	567.9904	C_18_H_7_F_14_O_3_P^+^	vw	4	EC_ox_

The data presented in Table 3 were obtained by conducting a GC-LRMS and GC-QTOF analysis. The objective of the GC-QTOF analysis was the detection of exact masses and the confirmation of the detection of the molecular ions of the TPs. To this end, a two-step process was undertaken. This involved the measurement with two different ionization energies: standard conditions (70 eV) and LE-EI (18 eV). LE-EI yielded lower fragmentation and confirmed the molecule ions of all TPs except for TP 9. The molecular ion of TP 9 (564.9928 m/z) could only be detected by LE-EI. Confidence in the screening results was increased when a TP was detected with multiple methods. One TP (TP 7) could be confirmed by a spectral library entry match. In addition, the CL provides further information on confidence and even isomeric possibilities.

**Fig. 3:**
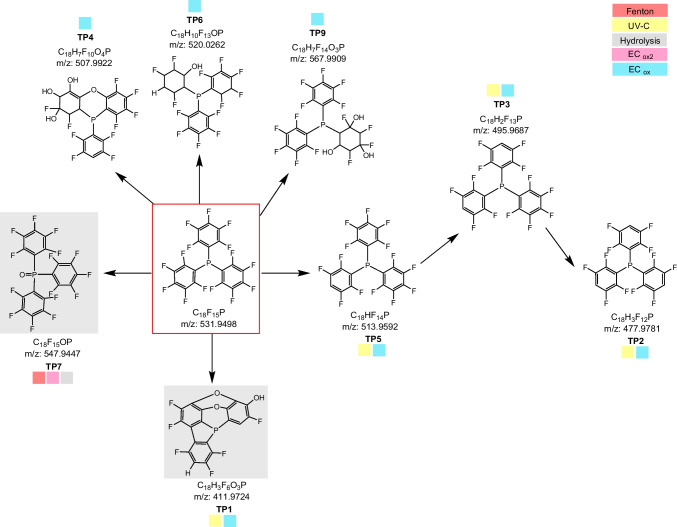
Proposed structures of the TPs from different simulation experiments of TPFP**P**, organized as possible transformation pathways (numbering refers to Table 3). Simulation method abbreviations: EC_ox_: NH_4_Ac; EC_ox2_: FA; gray background: indicates high occurrence

The applied simulation methods led to a relatively small number of TPs. However, some TPs were identified with relatively high intensities, especially TP 7 and TP 1. Once more, the TOP assay simulation method did not yield any TPs, suggesting that the substance had been completely decomposed by the conditions. Moreover, additional methods, such as alkaline hydrolysis and EC under reductive conditions, did not result in any changes in TPFP**P**. Consequently, no data are presented. Conversely, varying EC oxidation conditions yielded disparate outcomes as indicated by EC_ox_ and EC_ox2_. Figure 3 illustrates the proposed structures of the TPs and the proposed decomposition pathways. To exemplify the structures, a probable constitutional isomer was selected, given the fact that the EI spectra were not always able to provide unambiguous formulae for the derived TPs, as indicated by the CL.

#### Modifications

The generated TPs demonstrate oxidation on the phosphorous (TP 7) and several modifications on the aryl groups, including defluorination (e.g., TP 5, TP 3, TP 2), hydroxylation with and without fluorine release (e.g., TP 1, TP 4, TP 6, T 9), and diphenyl as well as diphenyl ether formation and dioxin structures (TP 1). TP 1, TP 4, TP 6, and TP 9 exhibit a multitude of modifications, whereas TP 4, TP 6, and TP 9 even experience a loss of the aromatic structures, possibly due to the hydrogenation of arene groups and water addition. The choice of the constitutional isomers of the TPs in Fig. 3 is based on an intriguing discovery. In phosphines, phosphorus(III) exhibits a positive mesomeric effect due to the free electron pair, which would therefore direct a substitution in the meta position. Nevertheless, the experimental data from Hanna (1977) with TPFP**P** yielded only products with para substitution. It is postulated that the overlap of the p-orbitals of the aryl ring and the empty orbitals of phosphorus stabilizes the negative charge on the phosphorus-bound carbon, resulting in para substitution [88]. TP 7, a phosphine oxide, is the only TP previously described [88, 89], and hence, it is confirmed by a spectral library entry. Given that phosphines are known to possess an oxophilic character, the formation of TP 7 is highly probable. The resulting product is expected to exhibit greater stability than the precursor, due to its inability to degrade further using the applied methods. The data indicate that the phosphine oxide exhibits even reduced reactivity, potentially attributed to a reduction in Lewis basicity. This emphasizes the necessity of eliminating aryl-bound fluorine for effective degradation.

#### Defluorination

However, defluorination is an important step in the overall decomposition process. Here, we report three TPs with specific defluorination, TP 5, TP 3, and TP 2, formed by UV-C irradiation and electrochemical oxidation. The proposed underlying mechanism is hydrodefluorination. This reaction is initiated by photoheterolytic cleavage and hydrogen abstraction in a polar solvent [59, 70, 71]. In addition to its environmental relevance, specific defluorination is also of importance with regard to partially fluorinated substances. These compounds are of great interest as agrochemicals and pharmaceuticals. This is because the synthesis of these partially fluorinated substances remains a challenging process, often requiring a complete fluorination followed by selective defluorination steps to obtain the desired product. The irradiation and EC methods reported here are therefore of interest for the synthesis of fluoroarenes [69, 90]. Another defluorination mechanism is indirect photolysis. Only TP 1, with a dioxin structure similar to that of TPFP**B**, was identified as the product of indirect photolysis (TP 16, TP 38).

#### Electrochemistry

The EC process yielded the greatest number of different TPs but required the use of elevated voltages. In contrast to the reductive electrochemical conditions, which did not yield reproducible products, the oxidative EC oxidation process resulted in the generation of reduction products. The observed outcome could suggest that the electrode and/or the conditions utilized are unsuitable for the reductive conversion of the precursor. Nevertheless, numerous different combinations of conditions (electrolytes, pH, solvents) did not alter the observed outcome. One possible explanation is that the high electron density within the aromatic structures and on phosphorus(III) impairs reactions under purely reductive conditions with a different underlying mechanism. Notably, alterations to the conditions within the EC (see SI-2 for a comprehensive enumeration of all EC parameters) did not lead to transformation under reductive conditions. Instead, the oxidative mode resulted in the formation of TP 7.

Consequently, the utilization of FA/acidic conditions results in a modification of the reaction and the formation of an oxidation product, whereas neutral conditions employing NH_4_Ac lead to defluorination and the generation of additional products. Some of the additional products with multiple modifications (TP 4, TP 6, and T 9) are formed exclusively with EC, albeit in low yields. While the proposed structures, based on the spectral analysis, are relatively plausible, their certainty is limited (CL = 4). In the absence of recognizable transformation pathways or precursors and no literature verification, it is uncertain whether these compounds have environmental significance, and annotation requires further confirmation. Nevertheless, the confirmation of all other TPs formed using EC demonstrates that EC is an effective screening approach for investigating TPs, as it is both simple and rapid.

Our results indicate a remarkable stability of the P–Ar bond, as evidenced by a resistance to cleavage even under conditions of high energy input. It is noteworthy that the hydrogenation of the arene groups, rather than the conventional bond cleavage, appears to be the primary mechanism responsible for the structural modification of these compounds. Potential competing pathways, including dehalogenation, hydrogenation, and hydroxylation, may contribute to the observed changes in chemical composition. These changes were detected even though the hydrogenation of arenes requires stringent conditions, which usually include a catalytic system.

#### Electrochemistry for biotic processes

For phosphines, the biotic transformation appears to differ from that of other substances, with limited literature evidence suggesting that phosphorus is exclusively oxidized (TP 7) in these reactions. This oxidation reaction, which is identified in this study as a predominant transformation, may also reflect biotic metabolism [91, 92]. This further supports the assumption that EC results can also be considered as a simulation of biotic transformation and, therefore, adds another important aspect to this study.

#### Exceptional stability

The transformation simulation of TPFP**P** yielded an unexpected result: its exceptional stability, despite the fact that phosphanes are strong oxophilic. This exceptional stability is evidenced by both the strict conditions necessary for its decomposition using the employed simulation techniques and the low minimal number of TPs observed. This stability can be attributed, in part, to its limited solubility in protic solvents, coupled with the focus of these methods on oxidative decomposition on the fluorinated ring, which is important to prevent the formation of stable intermediates. This process may be less favorable for a Lewis base with its free electron pair and therefore positive mesomeric effect, typically acting as an electron donor. This effect impairs a nucleophilic attack usually done by oxygen species on the fluorinated ring, which is already reduced in reactivity. This might explain the superior stability, especially in comparison to TPFP**B**. As a result, we categorize TP 7 as a possible “end product” and forever chemical of tomorrow. Even in the case of PFAS, which are commonly referred to as “forever chemicals” and consist of at least one fully fluorinated carbon, it is not necessarily the parent compounds that are “end products,” but the TPs.

## Conclusion

In this study, two FOCs (TPFP**B** and TPFP**P**) used as additives in LiB electrolytes were subjected to a series of methods (EC, UV-C, hydrolysis, Fenton) with the objective of simulating and identifying TPs. TPs were often identified through the application of diverse simulation methods, with the annotation of these compounds at varying confirmation levels [64]. Plausible degradation pathways were postulated, and for some TPs of TPFP**B** and TP 1 of TPFP**P,** information on structurally related compounds was found in the literature [65–68, 76, 88, 89, 93]. A novel EC transformation approach has been developed for substances with exceptional stability. This approach utilizes the Antec-EC system and conditions similar to those found in natural environments.

The TPFP**B** simulation yielded 49 distinct TPs, 28 of which could be annotated. Of these, 27 previously unreported annotated structures are presented here. The majority of these substances exhibit altered chemical behavior and stability in comparison to the precursor. Some of the products are structurally related to substances that have been classified as persistent organic pollutants (POPs) and have been identified as posing potential health risks [79]. Apart from that, the formation of oligomers was also observed. The electrochemical conversion of TPFP**B** results in the generation of a range of products that exhibit similarities to those produced by other simulation methods. In contrast to TPFP**B**, TPFP**P** may exhibit advantageous properties for LiBs due to its exceptional stability [56, 57], which is not environmentally favorable. The transformation of TPFP**P** was observed to occur under particularly rigorous conditions, which serves to underscore the substance's notable stability. Of greater concern is the observation that the eight identified TPs appear to be more stable than the precursor. One particular TP (TP 7), the only one that has been previously identified in the literature, exhibits high intensities in multiple simulations, thus warranting the classification of a “forever chemical.” Furthermore, the TPFP**P** EC simulation was capable of reproducing the complete set of TPs formed through the use of alternative simulation techniques. For both substances, consistent transformation pathways were identified, exhibiting analogous primary reactions but completely distinct TPs. The EC experiments were able to confirm all of the reactions that reflected abiotic processes, as well as the TPs suggested in the literature to reflect biotic processes in nature. TPFP**B** formed a highly toxic dioxin (TP 7) and potential dioxin-like structures (TP 4, TP 5, TP 6, and TP 8). In contrast, TPFP**P** itself is remarkably stable, resulting in the formation of an even more stable TP (TP 7). The hydrolysis results indicate the existence of a significant additional decomposition mechanism for anthropogenic substances present in the environment. This process should be considered when studying transformation pathways, despite the extreme conditions necessary for hydrolysis—conditions that do not reflect the environment—and the resulting low transformation rates. It can be concluded that these substances exhibit high levels of stability, and that hydrolysis represents a significant additional potential transformation path.

The identified TPs offer preliminary insights into the degradation pathways of TPFP**P** and TPFP**B**, which can be utilized for further investigations to gain deeper insight into the underlying mechanisms. Moreover, these findings establish a foundation for future toxicity studies, which are currently limited. This is particularly the case for fluorinated compounds and fluorinated analogues of already known POPs. Furthermore, these findings can be utilized to conduct targeted environmental analyses, thereby contributing to the enhancement of residue analysis in authentic samples derived from environmental sources or waste treatment effluents. It can be concluded that there is compelling evidence of the importance and complexity of the role of TPs in the context of effective risk assessment and providing first conclusive indications of environmental behavior for the assessment of the environmental relevance of FOCs.

## Supplementary Information

Below is the link to the electronic supplementary material.

Supplementary file1 (DOCX 392 KB)

## Data Availability

All data and material are available.
