# Supplementary material for: Identification of transformation products from fluorinated lithium-ion battery additives TPFPB and TPFPP: forever chemicals of tomorrow?
Source: Anal Bioanal Chem. 2024 Sep 13;416(28):6405–19. doi: 10.1007/s00216-024-05526-z (PMC11541385; doi:10.1007/s00216-024-05526-z)
Supplement: Supplementary file 1 — Supplementary file1 (DOCX 392 KB) [file 216_2024_5526_MOESM1_ESM.docx]

**Identification of transformation products from fluorinated lithium-ion battery additives TPFPB and TPFPP - forever chemicals of tomorrow?**

Juliane Scholl ^1,2^, Jan Lisec ^1^, Hajo Haase ^2^, Matthias Koch ^1,*^

*^1^ Bundesanstalt für Materialforschung und -prüfung (BAM); Department of Analytical Chemistry and Reference Materials*

*^2^ Technische Universität Berlin, Department of Food Chemistry and Toxicology*

* Correspondence:

Matthias Koch

E-Mail: matthias.koch@bam.de Telephone: +49 30 8104-71170

**SI-1 Detailed description of TPFPB data processing**

**R in-house script development**

The LC-QTOF data from the TPFPB simulation sets were pre-processed using MSDIAL for peak detection and spectra deconvolution. The exported result, a list of unknown compounds defined by unique combinations of exact masses (mz) and retention times (RT), was analyzed to identify potential TPs using a dedicated in-house script. Within the script compounds were identified as potential TPs when they fulfilled a set of criteria. Firstly, the intensity of the two replicate samples of the TP method was required to be 100-fold higher than the maximum obtained in solvent blanks, method blanks and control standards (control samples containing the parent compound but not subjected to any simulation steps). Secondly, defined minimum peak intensities (thr) were required in accordance with three retention time ranges: 1) RT 0 to 1.8 (thr = 5); 2) RT 1.8 to 8.05 (thr = 3.8); 3) RT 8.8 to 100 (thr = 4.5). In a third step, isotopic pattern of compounds was extracted from the raw data and used to support candidate selection as the boron isotopic pattern is highly specific. In order to select the molecular mass and calculate the chemical sum formula suggestions, the mass with the highest intensity in the isotope spectra was selected. In case of multiple deconvolutions of the same exact mass within a narrow RT window, a merge step was implemented (see Figure SI-1.1.).

**
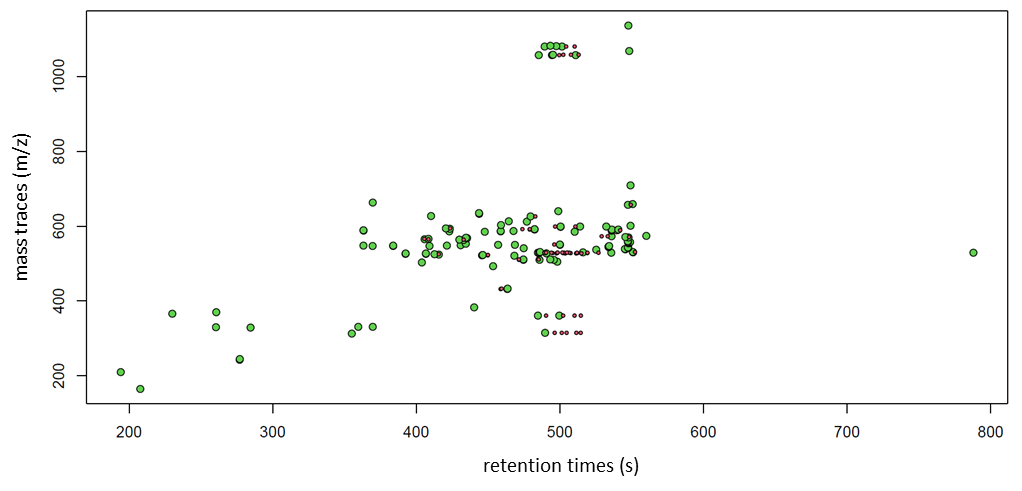
****Figure SI-1.1.** Example of visualization of data analysis using the developed R-script. The plot displays all unknown masses, matching the filtering criteria defined in the script, with red dots indicating highly repetitive masses (background traces, solvent) and green dots representing unique mz/RT pairs subjected to annotation in the next step. The mz intensities of unknowns are shown in dependence to the chromatographic RT in seconds (s).

Red dots in Fig.SI-1.1 represent the exact masses of the unknown components, which have been annotated repeatedly. The green dots represent the unknown components that remain after the merging step.

Compounds showing fragmentation in the ESI source might be represented as independent components in the MSDIAL result (deconvolution errors). To avoid redundant annotation, a RT grouping step was implemented, combining different mz which show a RT distance of less than 1.5 s, i.e. assuming that independent TPs are chromatographically separated by at least 1.5 s.


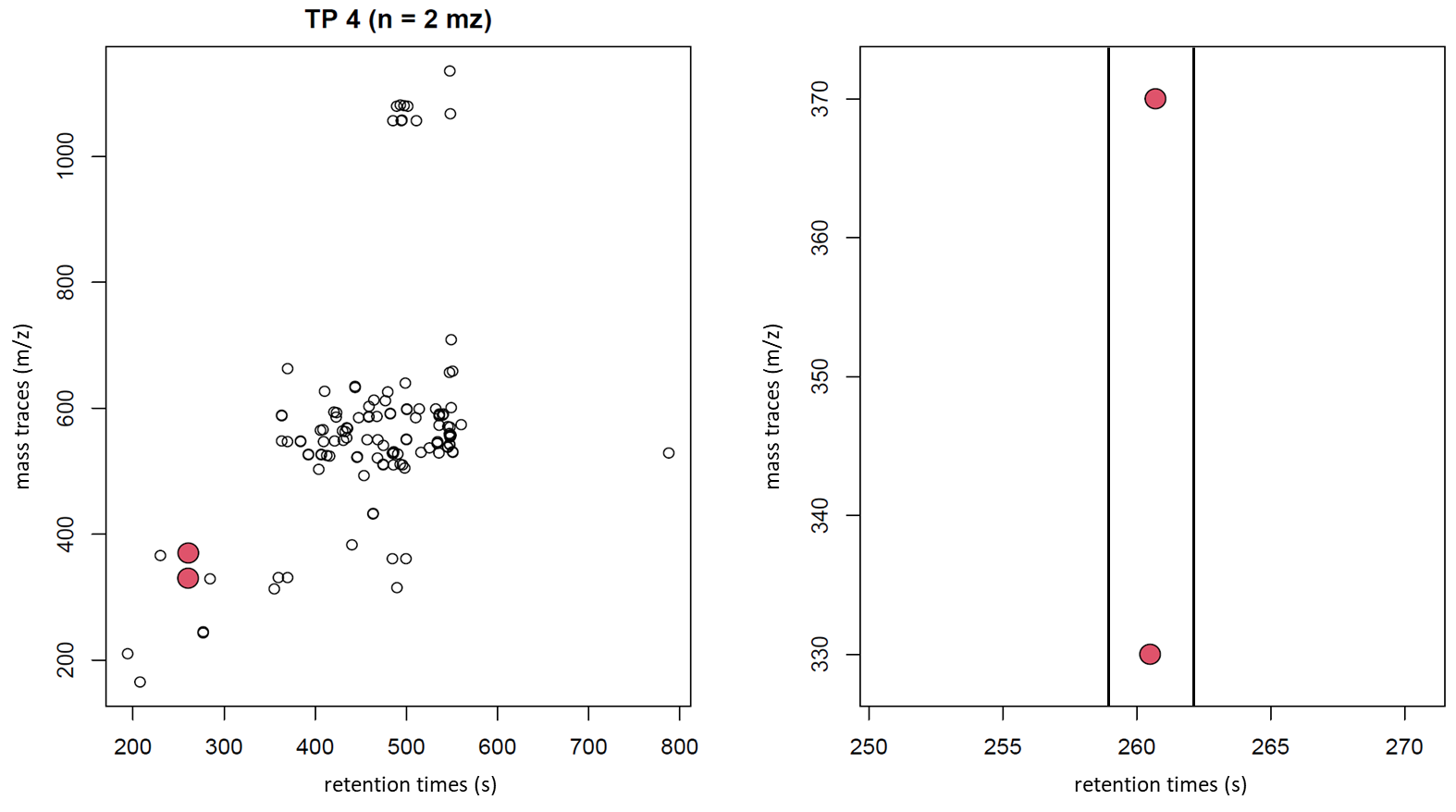


**Figure SI-1.2.** On the left, visualization of peaks grouped together as potential TP #4 (red dots) within the plot showing all candidate peaks after filtering. A zoomed-in version is on the right. Plotted are the unknown masses (m/z) in dependence to the chromatographic retention time in seconds (s).

The reliability of this peak grouping step was validated by analyzing chromatographic raw data of the grouped masses. Fragmentation was confirmed by matching chromatographic curves and mass ratios in all positive samples. An example of fragmentation is m/z 330.000 (Figure SI-1.3), which is identified as related to mass m/z 370.013.


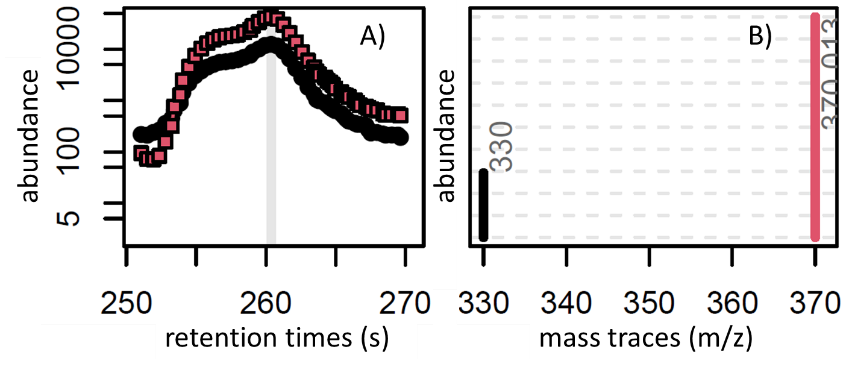


**Figure SI-1.3.** LC-QTOF raw data for mass traces (m/z) of a matching retention time group: **A)** abundance as a function of chromatographic retention time (s); **B)** mass spectrum of maximum intensity from A).

However, in 90% of cases, manual verification of the grouping step revealed no discernible relationship between the grouped mass traces and necessitating further manual verification (Figure SI-1.4., denser region).


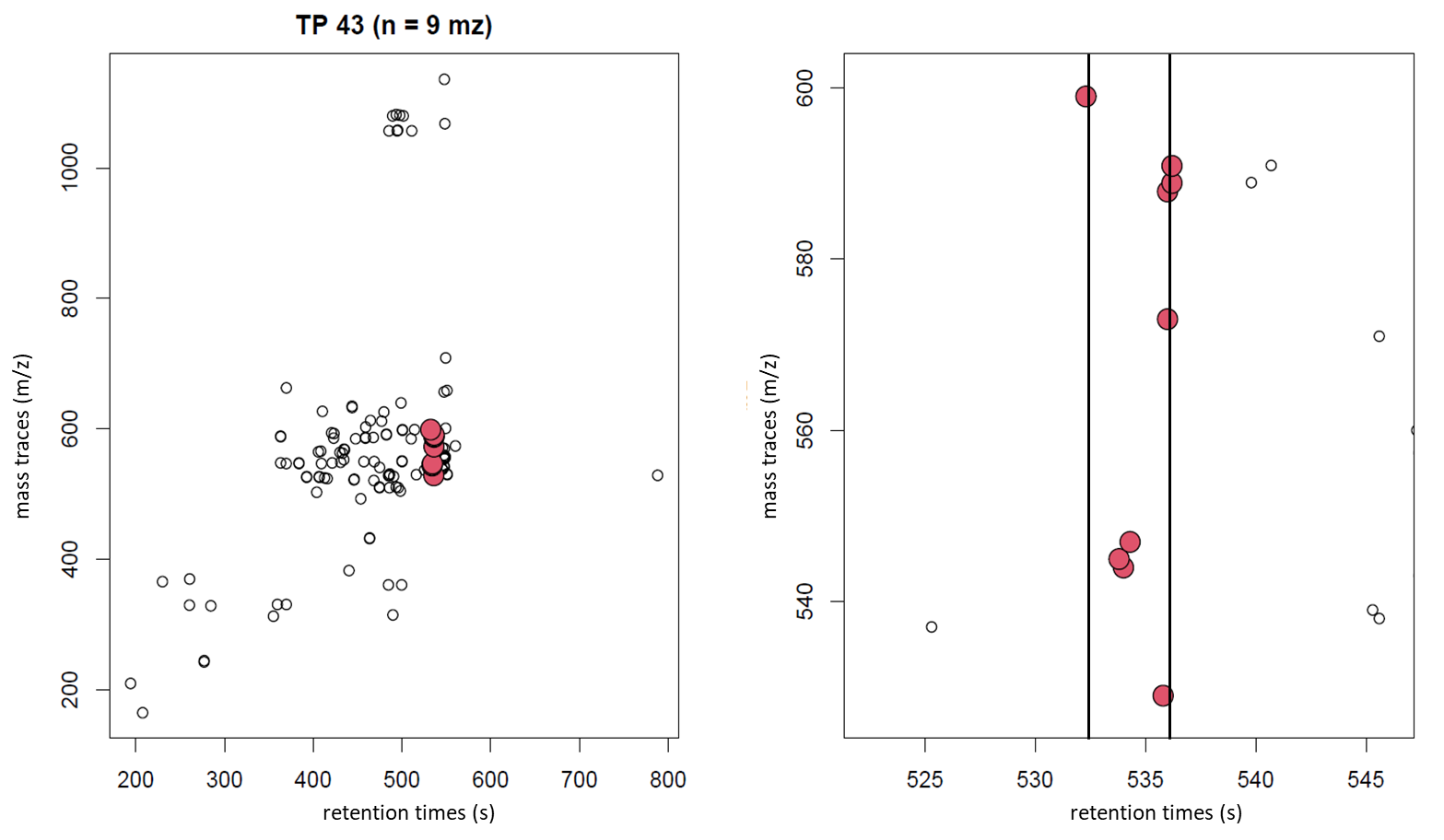


**Figure SI-1.4.** The figure on the left depicts a denser region of the grouping step with TP #43 (red dots) within the plot showing all candidate peaks after filtering. A zoomed-in version is presented on the right. The unknown masses (m/z) are plotted in dependence to the chromatographic retention time in seconds (s).

A manual analysis of the chromatographic raw data of the grouped masses (TP #43) revealed one matching chromatographic curve and mass ratio within all positive samples.

Suggestions for chemical sum formulas were calculated based on exact masses and restricted elements (C, F, N, H, O), with 5 rules implemented to minimize the number of inconclusive formulas based on elemental occurrence n_x_, where n is a positive integer and x a chemical element. The following rules were established to ensure the integrity of the data: (1) (n_F_+n_H_+n_O_)/n_C_>=0.55; (2) (n_F_+n_H_+n_O_ )/n_C_ <= 1.3; (3) n_N_ <= 1; (4) n_H_>=3 for n_N_>=1 and (n_F_/n_C_) > 0.4 (5). A threshold of ±3.5 mDA or ±3.5 ppm deviation was applied to the calculation of the TP sum formula. The mass spectrometric data, including isotopic abundances and MS/MS fragments of the remaining proposed molecular formulas, were subjected to a manual verification process to ascertain final structural formulas and the degree of confirmation (CL).

1. **R in-house script:**

The R-script utilized in this study can be made available upon request.

Pseudo script:

1. read in MSDIAL compound list (n=63 samples), MSDIAL result (m=4857 compounds in n=63 samples)
2. read in raw data of n=63 samples to later extract isotope pattern and generate QC plots
3. compound filtering for general peak quality/intensity
4. compound filtering for abundance in specific samples (replicate groups, m=189)
5. merge step (**time groups within mass groups**)
6. RT grouping step (m=127)
7. defining correct precursor_mass (**highest intensity mass in isotopic pattern**)
8. Calculation chemical sum formulas: threshold of ±3.5 mDA or ±3.5 ppm and application of elemental rules (see **SI-3.1.**)

**SI-2: Supplementary data on all EC experiments conducted to assess transformation.**

**Table SI-2.1**: A complete enumeration of all EC parameters utilized for the transformation of TPFPB and TPFPP

| Substance (conc.) | solvent | electrolyte | modifier | electrode | cell | potential range [V] | Detection |
| --- | --- | --- | --- | --- | --- | --- | --- |
| TPFPB (0.1 mM) | ACN/H_2_O (8:2 v/v) |  | 1%FA | Ag | Reactor | -2.5 to 3.5 | online |
| TPFPB (0.1 mM) | ACN/H_2_O (8:2 v/v) |  | 1%FA | BDD | μPrepCell | -2.5 to 3.5 | online |
| TPFPB (0.1 mM) | MeOH/H_2_O (8:1 v/v) |  | 1%FA | BDD | μPrepCell | -2.5 to 3.5 | online |
| TPFPB (0.1 mM) | MeOH/H_2_O (8:2 v/v) |  | 5 mM NH_4_Ac | Ag | Reactor | -2.5 to 3.5 | online |
| TPFPB (0.1 mM) | MeOH/H_2_O (8:2 v/v) |  | 5 mM NH_4_Ac | BDD | μPrepCell | -2.5 to 3.5 | online |
| TPFPB (0.1 mM) | MeOH/H_2_O (8:1 v/v) | TEABF4 | 5 mM NH_4_Ac | BDD | Reactor | -2.5 to 3.5 | online |
| TPFPB (0.1 mM) | MeOH/H_2_O (8:1 v/v) | 3.5 mM NH_4_OH |  | BDD | μPrepCell | -2.5 to 3.5 | online |
| TPFPB (0.1 mM) | ACN/H_2_O (8:1 v/v) | TBAP 1 mM |  | BDD | μPrepCell | -2.5 to 3.5 | online |
| TPFPB (10 µM) | ACN/H_2_O (99.7:0.3 v/v) |  | 1%FA | BDD | μPrepCell | -4.1 to 4.1 | online |
| TPFPB (10 µM) | ACN/H_2_O (99:1 v/v) |  | 1%FA | BDD | μPrepCell | -4.1 to 4.1 | online |
| TPFPP (1.5 mM) | ACN/H_2_O (8:2 v/v) |  | 1%FA | Ag | Reactor | -2.5 to 3.5 | offline |
| TPFPP (1.5 mM) | ACN/H_2_O (8:2 v/v) |  | 1%FA | BDD | μPrepCell | -2.5 to 3.5 | offline |
| TPFPP (1.5 mM) | MeOH/H_2_O (8:2 v/v) |  | 1%FA | Ag | Reactor | -2.5 to 3.5 | offline |
| TPFPP (0.5 mM) | MeOH/H_2_O (8:2 v/v) |  | 1%FA | BDD | μPrepCell | -2.5 to 3.5 | offline |
| TPFPP (1.5 mM) | MeOH/H_2_O (8:2 v/v) |  | 5 mM NH_4_Ac | BDD | μPrepCell | -2.5 to 3.5 | offline |
| TPFPP (0.5 mM) | MeOH/H_2_O (8:2 v/v) |  | 1% HAc | Ag | Reactor | -2.5 to 3.5 | offline |
| TPFPP (0.5 mM) | MeOH/H_2_O/Ac (4:1:2 v/v) |  | 5 mM NH_4_Ac | BDD | μPrepCell | -2.5 to 3.5 | offline |
| TPFPP (0.5 mM) | MeOH/H_2_O (8:1 v/v) | TBAP 1 mM |  | BDD | μPrepCell | -2.5 to 3.5 | offline |
| TPFPP (0.5 mM) | ACN/H_2_O (8:1 v/v) | TBAP 1 mM | 5 mM NH_4_Ac | BDD | μPrepCell | -2.5 to 3.5 | offline |
| TPFPP (0.1 mM) | MeOH/H_2_O (99:1 v/v) | TBAP 1 mM | 5 mM NH_4_Ac | BDD | μPrepCell | -2.5 to 3.5 | offline |
| TPFPP (0.02 mM) | MeOH/Ac (65:35 v/v) |  | 5 mM NH_4_Ac | BDD | μPrepCell | -4.0 to 4.0 | offline |
| TPFPP (0.02 mM) | ACN/EtOAc (65:35 v/v) |  | 1%FA | BDD | μPrepCell | -4.0 to 4.0 | offline |
| TPFPP (0.02 mM)) | ACN/EtOAc/H_2_O (65:35:0.3 v/v) |  | 1%FA | BDD | μPrepCell | -4.1 to 4.1 | offline |
| TPFPP (0.02 mM | MeOH/Ac (65:35 v/v) |  | 5 mM NH_4_Ac | BDD | μPrepCell | -4.1 to 4.1 | offline |

**SI-3: Supplementary data on TPs**

**Table SI-3.1.**: Compilation of the results of the different simulation methods employed on TPFPB using LC-QTOF (negative mode) data. The proposed chemical formulae, form of detection, intensities, number of formulas calculated with R script, number of formulas logical and coherent (manual check), annotation confirmation level (CL) and retention times are displayed for each compound. The intensities are indicated relative to the strongest signal, (vs: very strong) > 60%, (s: strong): 40–60%, (m: moderate): 20–40%, (w: weak) 10–20%, (vw: very weak) < 10%). Abbreviations of the simulation methods: EC (oxidative and reductive conditions); EC_ox_ (EC oxidative conditions); EC_red_ (EC reductive conditions); UV (UV-C irradiation); Fe (Fenton reaction); H (Hydrolysis with basic and acidic conditions); H_b_ (Hydrolysis alkaline conditions); H_a_ (Hydrolysis acidic conditions

| **TP-ID** | **MASS MEAS.** | **MASS CALC.** | **ERROR [mDA]** | **ERROR [ppm]** | **FORMULA** | **ADDUCT** | **FORMULAS CALC. R** | **FORMULAS CONCLUSIVE** | **INTENSITY** | **CL** | **SIMULATION METHOD** | **RETENTION TIME [min]** |
| --- | --- | --- | --- | --- | --- | --- | --- | --- | --- | --- | --- | --- |
| **1** | 164.9970 | 164.9969 | 0.1 | 1.0 | C_6_HF_4_O | [M-H]^-^ | 2 | 2 | vw | 5 | H_b_ | 3.46 |
| **2** | 209.9800 |  |  |  |  |  | 3 | 0 | vw |  | H_b_ | 3.24 |
| **3** | 244.9040 |  |  |  |  |  | 0 | 0 | vw |  | H_b_ | 4.62 |
| **4** | 312.9870 | 312.9905 | -3.5 | -11.2 | C_12_H_2_F_8_O | [M-H]^-^ | 3 | 1 | vw | 3 | H_b/_EC_red_ | 5.92 |
| **5** | 328.9820 | 328.9854 | -3.4 | -10.3 | C_12_H_2_F8O_2_ | [M-H]^-^ | 2 | 1 | vw | 5 | H_b_ | 4.74 |
| **6** | 330.9830 | 330.9811 | 1.9 | 5.7 | C_12_HF_9_O | [M-H]^-^ | 3 | 0 | vw | 5 | H_b_/EC_red_ | 5.99 |
| **7** | 366.0010 | 366.0007 | 0.3 | 0.8 | C_12_HF_7_O_3_ | [M+CH_3_CN-H]^-^ | 6 | 1 | vw | 5 | EC_red_ | 3.84 |
| **8** | 370.0050 | 370.0053 | -0.3 | -0.8 | C_12_H_2_F_8_O_2_ | [M+CH_3_CN-H]^-^ | 5 | 1 | vw | 5 | EC_red_ | 4,34 |
| **9** | 382.9910 | 382.9906 | 0.4 | 1.0 | C_12_BF_12_ | [M]^-^ | 7 | 2 | vw | 5 | EC_red_ | 7.34 |
| **10** | 432.9760 | 432.9771 | -1.1 | -2.5 | C_12_HBF_8_O_5_ | [M+HCOOH-H]^-^ | 13 | 1 | vw | 3 | EC | 7.73 |
| **11** | 493.0080 | 493.0075 | 0.5 | 1.0 | C_18_H_4_BF_13_O | [M-H]^-^ | 9 | 2 | vw | 4 | UV/H/Fe | 7.56 |
| **12** | 502.9790 |  |  |  |  |  | 0 | 0 | vw |  | EC_red_/EC_ox_ | 6.73 |
| **13** | 510.9970 | 510.9981 | -1.1 | -2.1 | C_18_H_3_BF_14_O | [M-H]^-^ | 9 | 2 | vw | 4 | UV/Fe/H | 7.91 |
| **14** | 520.9860 | 520.9860 | 0.0 | 0.0 | C_18_H_3_BF_12_O_4_ | [M-H]^-^ | 6 | 1 | vw | 4 | EC_ox_ | 7.81 |
| **15** | 522.9800 | 522.9817 | -1.7 | -3.3 | C_18_H_2_BF_13_O_3_ | [M-H]^-^ | 8 | 1 | vw | 4 | Fe/EC_red_/EC_ox_ | 7.43 |
| **16** | 524.9868 | 524.9834 | 3.4 | 6.5 | C_18_H_2_BF_9_O_5_ | [M+HCOOH-H]^-^ | 11 | 2 | vw | 3 | Fe/EC_red_/EC_ox_ | 6.88 |
| **17** | 525.0030 |  |  |  |  |  | 17 | 0 | vw |  | EC | 6.93 |
| **18** | 526.9920 | 526.9930 | -1.0 | -1.9 | C_18_H_3_BF_14_O_2_ | [M-H]^-^ | 9 | 2 | vw | 4 | Fe/H | 6.54 |
| **19** | 528.9870 | 528.9886 | -1.6 | -3.1 | C_18_H_2_BF_15_O | [M-H]^-^ | 9 | 2 | vs(EC_ox_)/ s(EC_red_) | 3 | Fe/H/UV/EC | 8.08 |
| **20** | 538.9940 | 538.9930 | 1.0 | 1.9 | C_18_HBF_14_ | [M+HCOOH-H]^-^ | 10 | 2 | vw | 3 | EC/UV | 9.09 |
| **21** | 541.0020 |  |  |  |  |  | 14 | 0 | vw |  | UV | 7.91 |
| **TPFPB+ MeOH** | 543.0040 | 543.0037 | 0.3 | 0.8 | C_18_BF_15_ | [M+H_3_COH-H]^-^ | 11 | 2 | w | 1 | UV | 9.12 |
| **22** | 547.0190 | 547.0192 | -0.2 | 0.6 | C_18_H_7_BF_14_O_3_ | [M-H]^-^ | 12 | 1 | vw | 4 | EC_red_ | 6.16 |
| **23** | 548.0030 |  |  |  |  |  | 10 | 0 | vw |  | EC_red_ | 5.40 |
| **24** | 548.0160 |  |  |  |  |  | 15 | 0 | vw |  | EC_ox_ | 6.40 |
| **25** | 548.9840 |  |  |  |  |  | 10 | 0 | vw |  | EC | 7.18 |
| **26** | 550.0090 | 550.0090 | 0.0 | 0.1 | C_18_HBF_14_O | [M+CH_3_CN-H]^-^ | 16 | 1 | vw | 4 | UV/EC_red_ | 7.62 |
| **27** | 550.9860 |  |  |  |  |  | 13 | 0 | vw |  | EC | 8.30 |
| **28** | 552.9940 |  |  |  |  |  | 15 | 0 | vw |  | EC_ox_ | 7.24 |
| **TPFPB+ FA** | 556.9880 | 556.9836 | 4.4 | 7.9* | C_18_BF_15_ | [M+HCOOH-H]^-^ | 13 | 1 | vw | 1 |  |  |
| **29** | 563.9990 |  |  |  |  |  | 9 | 0 | vw |  | EC/UV | 7.17 |
| **30** | 564.0090 | 564.0082 | 0.8 | 1.4 | C_18_H_2_BF_13_O_3_ | [M-H]^-^ | 16 | 1 | vw | 3 | EC | 7.22 |
| **31** | 566.0220 | 566.0239 | -1.9 | -1.4 | C_18_H_4_BF_13_O_3_ | [M+CH_3_CN-H]^-^ | 17 | 2 | vw | 3 | EC_ox_ | 6.76 |
| **32** | 569.0380 | 569.0388 | -0.8 | -2.1 | C_24_H_8_BF_13_O | [M-H]^-^ | 16 | 1 | vw | 3/4 | UV | 7.26 |
| **33** | 570.9980 |  |  |  |  |  | 11 | 0 | vw | 4 | Fe | 9.13 |
| **34** | 584.9870 |  |  |  |  |  | 18 | 0 | vw |  | EC | 8.51 |
| **35** | 586.0000 |  |  |  |  |  | 15 | 0 | vw |  | EC_ox_ | 7.05 |
| **36** | 586.9870 |  |  |  |  |  | 12 | 0 | vw |  | EC_ox_ | 7.65 |
| **37** | 590.9490 |  |  |  |  |  | 5 | 0 | vw |  | EC_ox_ | 9.00 |
| **38** | 592.0090 | 592.0067 | 2.3 | 3.9 | C_18_H_4_BF_11_O_7_ | [M+CH_3_CN-H]^-^ | 11 | 1 | vw | 3/4 | EC_ox_ | 8.04 |
| **39** | 594.0230 | 594.0224 | 0.6 | 1.0 | C_18_H_6_BF_11_O_7_- | [M+CH_3_CN-H]^-^ | 10 | 1 | vw | 3/2b | EC_ox_ | 7.06 |
| **40** | 602.9760 |  |  |  |  |  | 16 | 0 | vw |  | EC_ox_ | 7.65 |
| **41** | 612.0140 | 612.0129 | 1.1 | 1.8 | C_18_H_5_BF_12_O_7_ | [M+CH_3_CN-H]^-^ | 9 | 1 | vw | 3 | EC_ox_/Fe | 7.95 |
| **42** | 613.0220 |  |  |  |  |  | 11 | 0 | vw |  | Fe | 7.74 |
| **43** | 626.0100 |  |  |  |  |  | 15 | 0 | vw |  | EC_ox_ | 7.99 |
| **44** | 634.9350 |  |  |  |  |  | 1 | 0 | vw |  | H | 7.40 |
| **45** | 658.9830 | 658.9813 | 1.7 | 2.6 | C_24_H_5_BF_12_O_8_ | [M-H]^-^ | 14 | 1 | vw | 3 | Fe/UV/H | 9.18 |
| **46** | 662.9740 | 662.9726 | 1.4 | 2.1 | C_24_H_3_BF_14_O_6_ | [M-H]^-^ | 9 | 1 | vw |  | EC_red_ | 6.16 |
| **47** | 708.9640 |  |  |  |  |  | 10 | 0 | vw |  | EC_red_/Fe | 8.42 |
| **H_2_O adduct** | 1058.9851 | 1058.985 | 0.5 | 0.5 | C_36_H_4_B_2_F_30_O_2_ | [M-H]^-^ | 85 | 1 | vw |  | Fe/H/UV/EC | 8.24 |
| **dimer** | 1068.9660 | 1068.9689 | -2.9 | -2.7 | C_36_B_2_F_30_ | [M+HCOOH-H]^-^ | 86 | 1 | vw | 2b | Fe/H/UV/EC_ox_ | 8.04 |
| **48** | 1080.9560 | 1080.9550 | 1.0 | 0.9 | C_36_H_2_B_2_F_27_O_7_ | [M-H]^-^ | 92 | 1 | vw | 4 | Fe/H/UV | 8.16 |
| **49** | 1136.9640 |  |  |  |  |  | 99 | 1 | vw |  | EC | 9.13 |

**Table SI-3.2.**: The list of corresponding MS/MS data (m/z values) for the TPFPB TPs.

| **TP-ID** | **MASS MEAS.** | **MASS CALC.** | **MS/MS MASS MEAS.** | | | | | |
| --- | --- | --- | --- | --- | --- | --- | --- | --- |
| **1** | 164.9970 | 164.9969 |  |  |  |  |  |  |
| **2** | 209.9800 |  | 209.9800 | 163.9930 |  |  |  |  |
| **3** | 244.9040 |  |  |  |  |  |  |  |
| **4** | 312.9870 | 312.9905 | 312.9870 | 264.9864 |  |  |  |  |
| **5** | 328.9820 | 328.9854 |  |  |  |  |  |  |
| **6** | 330.9830 | 330.9811 | 285.0140 |  |  |  |  |  |
| **7** | 366.0010 | 366.0007 | 325.9860 | 303.9830 |  |  |  |  |
| **8** | 370.0050 | 370.0053 | 307.9900 | 261.9990 | 330.0000 |  |  |  |
| **9** | 382.9910 | 382.9906 | 165.0900 |  |  |  |  |  |
| **10** | 432.9760 | 432.9771 | 292.9770 |  |  |  |  |  |
| **11** | 493.0080 | 493.0075 | 166.9920 |  |  |  |  |  |
| **12** | 502.9790 |  | 258.9860 |  |  |  |  |  |
| **13** | 510.9970 | 510.9981 | 166.9910 | 174.9980 | 128.9950 |  |  |  |
| **14** | 520.9860 | 520.9860 | 474.9690 | 360.9860 | 324.9860 | 228.9890 | 156.9910 |  |
| **15** | 522.9800 | 522.9817 | 308.9780 | 258.9780 | 132.9900 |  |  |  |
| **16** | 524.9868 | 524.9834 | 166.9910 | 114.9990 | 140.9970 |  |  |  |
| **17** | 525.0030 |  | 163.0010 |  |  |  |  |  |
| **18** | 526.9920 | 526.9930 | 166.9890 | 144.9880 |  |  |  |  |
| **19** | 528.9870 | 528.9886 | 314.9850 | 360.9850 | 292.9821 |  |  |  |
| **20** | 538.9940 | 538.9930 | 166.9910 | 342.9920 |  |  |  |  |
| **21** | 541.0020 |  | 174.9970 |  |  |  |  |  |
| **TPFPB+ MeOH** | 543.0040 | 543.0037 | 166.9910 |  |  |  |  |  |
| **22** | 547.0190 | 547.0192 | 379.0220 |  |  |  |  |  |
| **23** | 548.0030 |  | 338.9820 |  |  |  |  |  |
| **24** | 548.0160 |  | 360.0000 |  |  |  |  |  |
| **25** | 548.9840 |  |  |  |  |  |  |  |
| **26** | 550.0090 | 550.0090 | 292.9800 |  |  |  |  |  |
| **27** | 550.9860 |  | 166.9920 | 280.9820 |  |  |  |  |
| **28** | 552.9940 |  | 163.0010 |  |  |  |  |  |
| **TPFPB+ FA** | 556.9880 | 556.9836 | 166.9910 |  |  |  |  |  |
| **29** | 563.9990 |  | 166.9910 | 314.9770 |  |  |  |  |
| **30** | 564.0090 | 564.0082 | 166.9910 | 314.9780 |  |  |  |  |
| **31** | 566.0220 | 566.0239 | 184.0170 | 239.2470 |  |  |  |  |
| **32** | 569.0380 | 569.0388 | 174.9970 | 166.9910 |  |  |  |  |
| **33** | 570.9980 |  | 166.9910 | 314.9850 |  |  |  |  |
| **34** | 584.9870 |  | 166.9910 |  |  |  |  |  |
| **35** | 586.0000 |  | 166.9930 | 570.9710 |  |  |  |  |
| **36** | 586.9870 |  | 166.9930 |  |  |  |  |  |
| **37** | 590.9490 |  |  |  |  |  |  |  |
| **38** | 592.0090 | 592.0067 | 166.9930 | 329.9980 |  |  |  |  |
| **39** | 594.0230 | 594.0224 | 360.0060 | 380.0120 | 166.9930 | 204.0270 |  |  |
| **40** | 602.9760 |  |  |  |  |  |  |  |
| **41** | 612.0140 | 612.0129 | 314.9860 | 166.9930 | 355.9900 |  |  |  |
| **42** | 613.0220 |  | 166.9900 |  |  |  |  |  |
| **43** | 626.0100 |  |  |  |  |  |  |  |
| **44** | 634.9350 |  | 272.9330 | 254.9840 | 174.0000 |  |  |  |
| **45** | 658.9830 | 658.9813 | 166.9900 |  |  |  |  |  |
| **46** | 662.9740 | 662.9726 | 330.9780 | 114.9370 |  |  |  |  |
| **47** | 708.9640 |  |  |  |  |  |  |  |
| **H_2_O adduct** | 1058.9851 | 1058.985 |  |  |  |  |  |  |
| **dimer** | 1068.9660 | 1068.9689 | 556.9790 | 360.9840 | 359.9910 | 314.9820 | 292.9800 | 166.9900 |
| **48** | 1080.9560 | 1080.9550 | 314.9810 |  |  |  |  |  |
| **49** | 1136.9640 |  |  |  |  |  |  |  |

**Table SI-3.3.**: Compilation of the results of the different simulation methods employed on TPFPP using GC-QTOF data. The proposed chemical formulae, intensities, number of formulas calculated, number of formulas logical and coherent (manual check), annotation confirmation level (CL) and retention times are displayed for each compound. The intensities are indicated relative to the strongest signal, (vs: very strong) > 60%, (s: strong): 40–60%, (m: moderate): 20–40%, (w: weak) 10–20%, (vw: very weak) < 10%). Abbreviations of the simulation methods: EC_ox_ (EC oxidative conditions: NH_4_Ac); EC_ox 2_ (EC oxidative conditions: FA); UV (UV-C irradiation); Fe (Fenton reaction); H_a_ (Hydrolysis acidic conditions). * TP molecule ion: 567.9928 (at 18 eV) and 554.9994 (at 70 eV)

| **TP-ID** | **MASS MEAS.** | **MASS CALC.** | **ERROR [mDA]** | **ERROR [ppm]** | **FORMULA** | **FORMULAS CALC. R** | **FORMULAS CONCLUSIVE** | **INTENSITY** | **CL** | **SIMULATION METHOD** | **RETENTION TIME [min]*** |
| --- | --- | --- | --- | --- | --- | --- | --- | --- | --- | --- | --- |
| **1** | 411.9731 | 411.9724 | 0.7 | 1.7 | C_18_H_3_F_6_O_3_P^+^ | 15 | 2 | w(EC_ox_)/ vw(UV) | 3 | EC_ox_/UV | 8.62 |
| **2** | 477.9796 | 477.9775 | 2.1 | 4.4 | C_18_H_3_F_12_P^+^ | 27 | 2 | vw | 3 | EC_ox_/UV | 10.09 |
| **3** | 495.9701 | 495.9681 | 2.0 | 4.0 | C_18_H_2_F_13_P^+^ | 32 | 2 | vw | 3 | EC_ox_/UV | 9.77 |
| **4** | 507.9901 | 507.9922 | -2.1 | -4.1 | C_18_H_7_F_10_O_4_P^+^ | 22 | 1 | vw | 3 | EC_ox_ | 11.16 |
| **5** | 513.9606 | 513.9587 | 1.9 | 3.7 | C_18_HF_14_P^+^ | 38 | 3 | vw | 3 | EC_ox_/UV | 9.47 |
| **6** | 520.0259 | 520.0256 | 0.3 | 0.5 | C_18_H_10_F_13_OP^+^ | 8 | 1 | vw | 4 | EC_ox_ | 11.20 |
| **TPFPP** | 531.9507 | 531.94926 | 1.44 | 2.7 | C_18_F_15_P^+^ |  |  | vs | 1 |  | 9.15 |
| **7** | 547.9453 | 547.9442 | 1.1 | 2.1 | C_18_F_15_PO^+^ | 51 | 2 | vs(EC_ox2_)/ vw(H_a_)/ vw(Fe) | 2a | Fe/EC_ox 2_/H_a_ | 10.02 |
| **8** | 555.9941 |  |  |  |  | 25 | 0 | vw |  | EC_ox_ | 11.47 |
| **9** | 567.9928* | 567.9904 | 2.4 | 4.3 | C_18_H_7_F_14_O_3_P^+^ | 25 | 1 | vw | 4 | EC_ox_ | 11.66 |

**Table SI-3.4.**: The following table presents the list of mass fragment values (m/z) derived from the TPFPP TPs spectra.

| **TP-ID** | **MASS MEAS.** | **MASS CALC.** | **MS FRAGMENTS** | | | | | | | | |
| --- | --- | --- | --- | --- | --- | --- | --- | --- | --- | --- | --- |
| **1** | 411.9731 | 411.9724 | 391.9658 | 381.9614 | 295.9871 | 264.9888 | 244.9790 | 68.9700 | 228.9840 | 214.9683 | 198.9730 |
| **2** | 477.9796 | 477.9775 | 328.9773 | 278.9791 | 260.0062 | 241.0073 | 111.0041 |  |  |  |  |
| **3** | 495.9701 | 495.9681 | 346.9681 | 277.9968 | 260.0055 | 258.9874 | 198.9730 | 179.9746 | 111.0041 | 99.0040 |  |
| **4** | 507.9901 | 507.9922 | 492.9644 | 377.0016 | 358.9877 | 315.9692 | 246.9980 | 179.9745 | 111.0041 | 92.0056 | 407.9989 |
| **5** | 513.9606 | 513.9587 | 364.9586 | 346.9681 | 277.9968 | 258.9874 | 226.9919 | 68.9700 |  |  |  |
| **6** | 520.0259 | 520.0256 | 505.0035 | 328.9778 | 260.0066 | 68.9701 | 241.0071 | 179.9745 | 111.0041 | 308.9698 |  |
| **TPFPP** | 531.9507 | 531.94926 | 364.9590 | 295.9876 | 276.9891 | 257.9906 | 226.9919 | 68.9700 |  |  |  |
| **7** | 547.9453 | 547.9442 | 380.9534 | 364.9591 | 295.9874 | 264.9884 | 216.9638 | 116.9947 |  |  |  |
| **8** | 555.9941 |  | 389.0010 | 364.9569 | 277.9955 | 191.0348 | 295.9871 | 128.9943 | 162.0086 |  |  |
| **9** | 567.9928* | 567.9904 | 526.9682 | 438.9982 | 364.9982 | 329.0014 | 216.9636 | 197.9654 | 162.0086 | 554.9997 |  |
